# Toward clarity: why we need to standardize the term ‘recurrent pregnancy loss’

**DOI:** 10.5935/1518-0557.20250158

**Published:** 2025

**Authors:** Luiza Pretto, Eduarda Nabinger, Ivan Sereno Montenegro, Maria Teresa Vieira Sanseverino, Osvaldo Artigalás, Fernanda Sales Luiz Vianna, Eduardo Pandolfi Passos, Lucas Rosa Fraga

**Affiliations:** 1 Universidade Federal do Rio Grande do Sul, Porto Alegre, RS, Brazil; 2 Hospital de Clínicas de Porto Alegre, Porto Alegre, RS, Brazil; 3 Pontifícia Universidade Católica do Rio Grande do Sul (PUCRS), Porto Alegre, Brazil; 4 Fertility Center, Hospital Moinhos de Vento (HMV), Porto Alegre, Brazil

**Keywords:** infertility, miscarriage, definition standardization, terminology harmonization

## Abstract

Recurrent pregnancy loss (RPL) is a complex reproductive condition that remains difficult to manage. Despite advances in identifying risk factors and treating cases with known causes, about half of the cases are still idiopathic, making effective treatment and prognosis challenging. In addition to clinical hurdles, a major issue in both the literature and medical community is the lack of standardization in terminology and precise definition for RPL. Here, we review the main discrepancies in current definitions and support adopting the term ‘recurrent pregnancy loss’ as the most appropriate and inclusive. We recommend recognizing RPL as a specific subtype of secondary infertility, characterized by two or more pregnancy losses. Standardizing this terminology is crucial for improving diagnosis, research comparison, and patient care in reproductive medicine.

## INTRODUCTION

Recurrent pregnancy loss (RPL) is an adverse reproductive outcome characterized by more than one pregnancy loss event. Although difficult to estimate, it affects 1-2% of women worldwide (ESHRE Guideline Group on RPL *et al.*, 2023). There are several risk factors for RPL, including genetic causes such as parental chromosomal abnormalities and embryonic aneuploidies; anatomical factors like congenital uterine malformations, intrauterine adhesions, and myomas; immunological disorders, including antiphospholipid antibody syndrome, hereditary and acquired thrombophilias, and autoimmune diseases; and endocrine factors such as polycystic ovary syndrome, thyroid dysfunction, and hyperprolactinemia (Rai & Regan, 2006; Passos *et al.*, 2023). Other associated conditions include advanced maternal age and lifestyle habits, particularly smoking, excessive alcohol or caffeine intake, illicit drug use, exposure to toxins or radiation, and chronic stress (Ng *et al.*, 2021; Sun *et al.*, 2023). More recently, male factors such as high sperm DNA fragmentation have also been added to the list of RPL risk factors (Tan *et al.*, 2019; Inversetti *et al.*, 2025). Despite the broad range of risk factors and causes, they do not cover all possible etiologies related to the condition. In this context, it is estimated that approximately half of the cases remain unexplained (Saravelos & Regan, 2014; ESHRE Guideline Group on RPL *et al.*, 2023). Due to the high number of idiopathic cases, RPL requires ongoing efforts by the scientific community.

Additionally, a key issue emerges at both the research and clinical levels: the inconsistent use of numerous terms and descriptions. While it remains challenging to determine whether this lack of uniform terminology results from divergent scientific studies or from the absence of standardization among professional societies - or vice versa - the need to clarify this situation has become increasingly clear. This inconsistency impedes the comparison of study results, introduces ambiguity in diagnosis, and makes developing evidence-based clinical protocols more difficult. Therefore, standardizing terminology is crucial - not only to enhance communication among professionals and ensure accurate patient care, but also to strengthen the scientific rigor of research and clinical practice (Rabin *et al.*, 2012; Chae-Kim *et al.*, 2023). A unified term and a consistent definition would enable more accurate data collection, improve identification of etiological patterns, and support more coherent strategies for the prevention, diagnosis, and treatment of RPL. Understanding and harmonizing terms and definitions is not just an academic concern but a vital step toward ensuring research accuracy and implementing more effective and equitable treatments.

## DEFINITION AND CLASSIFICATION

Throughout biomedical literature, various terms describe cases of experiencing more than two pregnancy losses, such as “recurrent pregnancy loss,” “recurrent miscarriage,” “recurrent abortion,” “repeated pregnancy loss,” “recurrent spontaneous abortion,” “multiple miscarriages,” “chronic pregnancy loss,” and others. Among these, “recurrent pregnancy loss” is the most comprehensive and neutral term, covering losses at any stage of pregnancy without negative connotations. Conversely, “recurrent miscarriage” usually refers to early pregnancy losses before 20 weeks and does not include later stages. Terms like “recurrent spontaneous abortion” and “recurrent abortion” are more traditional but can carry emotional and social stigma because of the word “abortion,” which may cause discomfort for patients. More descriptive terms such as “multiple miscarriages” or “repeated pregnancy loss” lack formal clinical definitions and may be used inconsistently, while “chronic pregnancy loss” is generally avoided because it incorrectly suggests a persistent condition rather than episodic events.

Due to the lack of standardization, the use of terms becomes arbitrary, as the choice ultimately rests with the individual conveying the information. Not surprisingly, an Irish study on the qualitative perception of RPL among healthcare professionals and patients highlighted the lack of a clear definition of the term in both groups, supporting our concern (Dennehy *et al.*, 2022). This inconsistency impacts the design and interpretation of clinical studies, which are crucial for changing the unfavorable idiopathic statistic. Although the terms essentially refer to the same condition, differences can be identified among studies. A similar trend is seen in the official documents of leading reproductive medical societies (Table 1). When analyzing the guidelines, it is clear that the main discrepancies are primarily in: 1) the number of lost pregnancies considered; 2) whether these are consecutive events or not; and 3) whether the occurrence should be regarded as infertility.

**Table 1:** How leading reproductive medicine societies name and define multiple pregnancy loss events.

Society	Nomenclature	Criteria	Recognized as infertility?	Reference
American College of Obstetricians and Gynecologists (ACOG)	Recurrent pregnancy loss	Two or three or more consecutive pregnancy losses	Not mentioned	American College of Obstetricians and Gynecologists, 2002
American Society for Reproductive Medicine (ASRM)	Recurrent pregnancy loss	The spontaneous loss of two or more pregnancies	No	Practice Committee of the American Society for Reproductive Medicine, 2012 Practice Committee of the American Society for Reproductive Medicine, 2020
European Society of Human Reproduction and Embryology (ESHRE)	Recurrent pregnancy loss	The loss of two or more pregnancies	Not mentioned	ESHRE Guideline Group on RPL *et al*., 2023
Nederlandse Vereniging voor Obstetrie en Gynaecologie (NVOG)	Recurrent miscarriage	Three or more consecutive pregnancy losses	Not mentioned	Goddijn *et al*., 2008
Royal College of Obstetricians and Gynaecologists (RCOG)	Recurrent miscarriage	Three or more first trimester miscarriages	Not mentioned	Regan *et al*., 2023

## THE NUMBER OF LOST PREGNANCIES

The number of pregnancy losses needed for diagnosis is the first key difference observed (Table 1). There is a consensus in the biomedical literature and among leading societies that a single pregnancy loss should not prompt investigation, as it can be considered an isolated and common event likely caused by known risk factors mentioned earlier. Conversely, there is agreement that waiting until a couple has experienced four or more pregnancy losses before investigation is a waste of time and reflects a lack of medical commitment to patients (Sierra *et al.*, 2025). With this in mind, the main inconsistency lies between considering two or three losses for the definition of RPL. Interestingly, a retrospective study of 383 couples experiencing pregnancy loss showed that the associated risk factors are similar between those with two and three or more losses, with no significant differences (Youssef *et al.*, 2020). These findings were supported by a systematic review and meta-analysis, as well as a comparative study, which concluded that the prevalence of abnormal results in diagnostic tests did not differ significantly between women experiencing two or three losses (Jaslow *et al.*, 2010; Van Dijk *et al.*, 2020). Additionally, a clinical study by Bashiri *et al.* (2012) demonstrated that, although slight variations in laboratory parameters were observed, these differences did not lead to significant disparities in pregnancy outcomes. Therefore, it appears that two losses already warrant attention from healthcare professionals and should be investigated. A growing consensus supports that clinical investigation after two pregnancy losses is not only justified but desirable, as it anticipates diagnosis, improves early intervention, and reduces the physical, emotional, and financial suffering of affected couples (Sierra *et al.*, 2025). This view is reinforced by partial overlap between RPL evaluation and the diagnostic process for other infertility causes, as well as by the increasing prevalence of delayed childbearing, which heightens the urgency of the diagnostic process.

## THE CHRONOLOGY OF EVENTS

Regarding the chronology of events, two points must be considered. The first involves the confusion around the use of the words ‘recurrent’ and ‘consecutive.’ It is important to note that ‘recurrent’ refers to something that happens repeatedly over time but not necessarily continuously, whereas ‘consecutive’ implies immediate succession, with one event following directly after another without interruption. The second point discusses the difference between primary RPL (pRPL) and secondary RPL (sRPL), which relate to the woman’s history of live births. pRPL is defined as all pregnancies ending in loss (spontaneous or stillbirth). Conversely, sRPL is understood as occurring after at least one live birth (Boedeker *et al.*, 2023). This classification helps clarify the causes of RPL. While pRPL appears to be more related to genetic causes, sRPL tends to be associated with acquired causes such as thrombophilia, infections, or postpartum uterine changes (Tersigni *et al.*, 2025).

Relevant clinical and prognostic differences between pRPL and sRPL have already been documented in the literature. In a population-based study, Shapira *et al.* (2012) observed that women with a prior history of pRPL had a higher incidence of preterm birth, fetal growth restriction, and gestational diabetes compared to those with sRPL. This indicates that different underlying mechanisms may be involved in pRPL and sRPL. Given this evidence, accurate classification and proper use of these terms are crucial for effectively guiding etiological investigations and reproductive counseling. In our view, considering the available labels and the distinction between pRPL and sRPL, it is essential to acknowledge that a healthy pregnancy following losses neither excludes a previous history nor reliably predicts favorable outcomes in future offspring of the couple. This is especially relevant because virtually none of the known etiological factors-genetic, anatomical, immunological, or endocrine-imply a 100% risk of loss, meaning a normal pregnancy cannot serve as a definitive indicator to differentiate between pRPL and sRPL. Furthermore, whether the events are consecutive or not, they still fit within the clinical condition described here. Since the concept of temporality seems to function more theoretically than practically, we recommend using the term ‘recurrent’.

## THE RECOGNITION AS INFERTILITY

Infertility is a condition characterized by a couple’s inability to establish a clinical pregnancy after one year (for women up to 35 years old) or six months (for women over 35 years old) of regular attempts to conceive without using contraceptive methods (Zegers-Hochschild *et al.*, 2017a, 2017b). It can be classified as primary, when the couple has never experienced previous pregnancies, and secondary, when the woman has had at least one prior pregnancy, regardless of the outcome, but is unable to conceive again afterward (Zegers-Hochschild *et al.*, 2017a, 2017b; Vander Borght & Wyns, 2018). From this categorization, RPL should be considered a form of infertility, particularly secondary infertility, especially if it has been more than 12 months since the last pregnancy loss. It shares a common characteristic with many other conditions - ultimately preventing a couple from having a live-born child. Notably, couples experiencing RPL often also face difficulties earlier in the process, during attempts to conceive within the period considered “normal.” Furthermore, the investigative approaches for RPL and infertility are similar. Therefore, RPL should not be considered in isolation from the broader concept of infertility. Recognizing RPL within this spectrum is crucial not only for appropriate medical management but also to ensure that couples facing this condition receive the comprehensive care, attention, and support they deserve in their journey toward parenthood.

## THE MALE ISSUE

To go further, the discussion can be expanded to whether pregnancy losses occurred within the same couple. Infertility is often still stigmatized, and treatment mainly focuses on women. As already shown here, men are also a vital part of the equation. In this context, it is also unclear whether studies on RPL consistently consider this variable in their design. While there is no doubt that a woman’s previous obstetric events are clinically essential and must be taken into account, should the definition of the concept only consider events between the same couple, or also include previous events of both partners? Can these different situations be combined, or is this sum (losses from previous relationships) insufficient for etiological investigation? This remains an open question, and to the best of our knowledge, it has not been discussed in the literature.

## CONCLUSION

All the points mentioned highlight a critical issue that must be addressed: research studies in this field are often flawed, limiting our ability to better understand the condition and treat patients. Due to the lack of standardization in terminology and conceptual understanding, RPL studies are heterogeneous. This heterogeneity occurs both within the same study, where patients are grouped together despite being clinically distinct, and among different studies, due to the lack of criteria in the use of the term. Additionally, systematic reviews and meta-analyses are also hindered for the same reasons. The absence of standardization of a single term or description is just the tip of the iceberg for progress in this area, and the consequences are immeasurable. We may be failing in our decision-making.

To sum up, it is clear that there is an emerging need to standardize the definition and use of the term ‘recurrent pregnancy loss’ to ensure clear communication and consistency in the diagnosis and treatment of the outcome described here. In our opinion, the best way to conceptualize RPL is as “secondary infertility characterized by the occurrence of two or more pregnancy losses.” We strongly advocate for unified and proactive efforts across scientific communities to standardize terminology and improve outcomes for couples who rarely suffer from the disorder.
